# Collaborating in the Time of COVID-19: The Scope and Scale of Innovative Responses to a Global Pandemic

**DOI:** 10.2196/25935

**Published:** 2021-02-09

**Authors:** Theresa Bernardo, Kurtis Edward Sobkowich, Russell Othmer Forrest, Luke Silva Stewart, Marcelo D'Agostino, Enrique Perez Gutierrez, Daniel Gillis

**Affiliations:** 1 Department of Population Medicine University of Guelph Guelph, ON Canada; 2 Information Systems for Health, Evidence and Intelligence for Action in Health Pan American Health Organization and World Health Organization Regional Office for the Americas Washington, DC United States; 3 Health Information and Risk Assessment Unit Pan American Health Organization and World Health Organization Regional Office for the Americas Washington, DC United States; 4 School of Computer Science University of Guelph Guelph, ON Canada

**Keywords:** crowdsourcing, artificial intelligence, collaboration, personal protective equipment, big data, AI, COVID-19, innovation, information sharing, communication, teamwork, knowledge, dissemination

## Abstract

The emergence of COVID-19 spurred the formation of myriad teams to tackle every conceivable aspect of the virus and thwart its spread. Enabled by global digital connectedness, collaboration has become a constant theme throughout the pandemic, resulting in the expedition of the scientific process (including vaccine development), rapid consolidation of global outbreak data and statistics, and experimentation with novel partnerships. To document the evolution of these collaborative efforts, the authors collected illustrative examples as the pandemic unfolded, supplemented with publications from the JMIR COVID-19 Special Issue. Over 60 projects rooted in collaboration are categorized into five main themes: knowledge dissemination, data propagation, crowdsourcing, artificial intelligence, and hardware design and development. They highlight the numerous ways that citizens, industry professionals, researchers, and academics have come together worldwide to consolidate information and produce products to combat the COVID-19 pandemic. Initially, researchers and citizen scientists scrambled to access quality data within an overwhelming quantity of information. As global curated data sets emerged, derivative works such as visualizations or models were developed that depended on consistent data and would fail when there were unanticipated changes. Crowdsourcing was used to collect and analyze data, aid in contact tracing, and produce personal protective equipment by sharing open designs for 3D printing. An international consortium of entrepreneurs and researchers created a ventilator based on an open-source design. A coalition of nongovernmental organizations and governmental organizations, led by the White House Office of Science and Technology Policy, created a shared open resource of over 200,000 research publications about COVID-19 and subsequently offered cash prizes for the best solutions to 17 key questions involving artificial intelligence. A thread of collaboration weaved throughout the pandemic response, which will shape future efforts. Novel partnerships will cross boundaries to create better processes, products, and solutions to consequential societal challenges.

## Introduction

COVID-19, caused by the transmission of SARS-CoV-2, was declared a pandemic in March 2020. In the early days of the COVID-19 pandemic, Karl Gude, former director of Infographics at Newsweek magazine, wanted to use his talents to help the public protect themselves against the onslaught of the new virus. He collaborated with a nurse and an epidemiologist, using Centers for Disease Control and Prevention resources [1] as a base to create the infographic “Break the Chain of Infection,” which was widely circulated on social media in March 2020 [2]. In response to demand, translations were crowdsourced into Arabic, Chinese, French, German, Italian, Japanese, Korean, Malayalam, Portuguese, and Spanish [3]. The United Nations subsequently called upon the global power of creatives to help stop the spread of COVID-19 [4,5].

Scientists have also taken advantage of unprecedented capabilities for global information sharing to accelerate their research. China sequenced the genome of the virus and shared it with the world on January 12, 2020, igniting investigation into the characteristics of the virus and enabling development of diagnostic tests. The gene sequence data was submitted for posting on Virological [6], a hub for prepublication data [7].

Similarly, in the race to combat the pandemic, researchers have posted their preliminary findings as preprints for global online scrutiny in advance of peer review and journal publication. The two most popular preprint servers, bioRxiv and medRxiv, posted nearly 3000 COVID-19 studies by May 7, 2020 [8]. Nature launched an open-source platform, The Outbreak Science Rapid PREreview, which allows scientists with ORCID IDs to submit their reviews as they read the preprints [9]. A new journal from MIT Press, Rapid Reviews: COVID-19, will use artificial intelligence (AI) to categorize preprints by discipline, novelty, and importance prior to review by humans [10]. Innovation to expedite review processes also includes peer-reviewed journals. A preprint analysis of 14 medical research journals found that average turnaround times had fallen from 117 to 60 days [8].

The JMIR journals, which are fully online, have set a precedent by partnering with the World Health Organization (WHO) on an e-collection *Theme Issue 2020: Novel Coronavirus (COVID-19) Outbreak Rapid Reports*, giving priority to the review and publication of COVID-19 articles on an ongoing basis [11]. The JMIR COVID-19 Special Issue citation library can be easily accessed via its download button. As of July 28, 2020, it included 147 papers that were screened by two of the authors to identify relevant references to supplement examples of collaboration collected by the authors throughout the pandemic, as well as through personal experience gained through the development of a COVID-19 dashboard for Canada [12].

The objective of this paper is to provide an illustrative, rather than exhaustive, overview of the range and types of collaboration stimulated by COVID-19 on a variety of fronts (research, public health information, personal protective equipment [PPE] shortages, etc) and involving individuals, academia, governments, and the private sector (Table 1). Finding the right partners for collaboration may require reaching outside existing networks to add complementary skills and perspectives [13]. Case in point, several of the authors of this paper have never met each other in person. Although forming successful new relationships entails risk and cost, it can also unlock creative potential to confront complex challenges [13].

**Table 1:** Overview of the scope and scale of collaboration to combat COVID-19.

Section		Scale of collaboration	Outcomes
Knowledge dissemination		Global	Multilingual infographics >40 million shares of cocreated articles COVID-19 eLearning
**Data**			
	The collection, and dissemination of COVID-19 data	Global	Dashboards and data visualization
	Sharing data among countries in the Americas	International (Western Hemisphere)	Data sharing agreements/policies
**Crowdsourcing**			
	The power of the crowd	Global	Hackathons Software development Hive mentality problem solving
	Crowdsourcing and telemedicine	Global	Communicate, work, interact, share information, and generate and exchange knowledge
	Crowdsourcing and artificial intelligence	Global	>200,000 articles in shared data set available for data and text mining
**Artificial intelligence**			
	Predicting the spread of a global pandemic	Global	Predictive models assessing international transmission
	Predicting those most vulnerable	Global	Predictive models assessing those most at risk
**Hardware**			
	Personal protective equipment	National^a^ (Canada)	10 million face shields 90,000 medical gowns
	Ventilators	International^a^ (Canada, US, Italy)	10,000 medical ventilators
	Test development and deployment	Global	Noninvasive saliva-based antigen test for COVID-19

^a^This refers to the examples used in the text; the World Health Organization distributed these items worldwide.

## COVID-19 Public Health Information and Education

The quantity of information on COVID-19 available to the public on virtually every communication channel can be truly overwhelming, while quality varies widely from peer-reviewed research to purposeful misinformation [14]. Even renowned publications such as the Economist or the Atlantic, whose content is usually behind a paywall, have provided free access to their COVID-19 coverage [15]. On February 15, 2020, the WHO recognized that the COVID-19 outbreak was accompanied by an infodemic (an overabundance of information, only some of which is accurate) and created a framework to manage it based on an online, crowdsourced technical consultation [16].

Amid the tsunami of information, a remarkable and highly successful example of collaboration to inform the public was initiated by Tomas Pueyo, a French and Spanish writer, engineer, and businessman based in San Francisco. Familiar with exponential growth rates, he published a data-driven article on Medium titled “Coronavirus: Why You Must Act Now” on March 10, 2020, which was viewed by 40 million people and translated into 30 languages before the month ended [17].

Collaboration can take many forms. It may be the stated intent from the onset or can be stimulated by the actions of an individual. Tomas later said, “I’m nobody. I’m just a guy in the right place and right time with enough storytelling ability and analytics ability to put it together” [17]. He sounded the alarm in a manner that resonated with many. Others took up the call. By March 14, 2020, the Khan Academy, a nonprofit that aims to provide free world-class education [18], had produced a video with a detailed explanation of concepts from Pueyo’s analysis [19]. Pueyo’s [20] second Medium post on March 19 was described as a team effort involving over a dozen normal citizens working around the clock to find all relevant research and structure it into a cohesive whole.

Among the numerous online courses about COVID-19, FutureLearn had 233,575 participants enrolled as of August 2020 [21]. There are also highly specialized courses to strategically encourage expert collaboration, such as Johns Hopkins’s course on epidemiology for data scientists [22] or Stanford’s course on COVID-19 and AI [23].

## Data

### The Collection and Dissemination of COVID-19 Data

Managing and responding to a pandemic requires getting the right information to the right people at the right time and in the right format. In times of crisis, however, quality and disaggregated data are not always readily available from standard sources such as public health or other governmental agencies. Data-sharing policies and lack of transparency might also interfere with the ability to conduct timely analyses [24,25]. In the case of SARS-CoV-2, the inability to rapidly collect robust public health data and share information on the spread of the pandemic could have constrained the advance of scientific knowledge. In particular, it could have affected the immediacy of care, the availability of PPE for frontline practitioners, and the success of public health mitigation strategies.

In response to the lack of public information in the early stages of the COVID-19 pandemic, citizens and academics took to scraping and compiling data from the internet. In early January 2020, Avi Schiffman, a then 17-year-old high school student from Washington State, United States, published a website to track the disease as it moved around the world. His site was viewed by over 2 million unique visitors by March 3 [26], growing to 3 million in another 8 days, demonstrating the public’s appetite for such information [27].

Interestingly, when data began appearing on government websites, they were not presented in a consistent manner, and as the pandemic continued, there were changes in the types of data reported and the manner in which they were provided. As local, regional, and federal public health agencies came to grips with the reality of the pandemic, their data became more readily available, interoperable, and consistent (although not without discrepancies and challenges [28]). Official Canadian data published on the Government of Canada websites, for example, were not downloadable until March 30, 2020, and the list of variables available changed over time. Depending on the source of information (ie, federal or provincial) and the time of the query, the data could have been presented in aggregate form with new or cumulative cases; with or without case status; and possibly with age, gender, race, or socioeconomic status [29]. These changes meant that anyone attempting to automate analyses and visualizations would be frequently forced to update their web scrapers and code.

When waiting for improvements to provincial and federal repositories to better understand how SARS-CoV-2 was spread, researchers and journalists resorted to using the Wayback Machine to access archived snapshots of pandemic data, including journalists at Maclean’s magazine who used archived data to generate a pandemic curve of daily new cases [30]. Researchers at the University of Toronto, University of Guelph, and The Hospital for Sick Children (SickKids) developed a Canadian repository of data by painstakingly scraping media sources for details about COVID-19 cases [29]. In the United States, researchers at Johns Hopkins have been at the forefront, compiling and managing a global data set of pandemic metrics [31]. Updated daily, it has been used by numerous organizations for modeling, visualization, and knowledge mobilization [31]. Other online data sources include the Centers for Disease Control and Prevention, Worldometer, and Our World in Data (to name a few).

To make these data meaningful to the public, researchers and citizen scientists published online dashboards to visualize various aspects of the pandemic, including (but not limited to) spread, daily and cumulative case and mortality counts, hospitalization rates, testing, doubling times, and positive case rates [12,32,33]. Some of the earliest dashboards were designed by the researchers behind the Johns Hopkins data set [31] and the team of University of Toronto, University of Guelph, and SickKids researchers behind the Canadian COVID-19 data set [32]. These dashboards are subsequently addressed in the “Crowdsourcing” section. Developers, however, had to design or redesign their dashboards based on how the data that supported them were obtained (ie, manually collected and managed; automatically pulled using web scrapers; or directly accessed through downloadable files, database queries, or application programming interface statements). The COVID-19 Dashboard in Canada [12] required several updates to account for changes in the formats and types of data available as the pandemic progressed.

Although there is a danger that errors may be propagated, the open nature also serves as a safeguard, as others can discover errors with fresh eyes. For example, a reanalysis of code and data found that presymptomatic infections are spread over a longer time period before symptom onset than previously thought. Thus, tracing contacts from 2 or 3 days before symptom onset may not be sufficient to find all secondary cases. Detection of this error was only possible because the original code and data were available and accessible, and the authors noted that it was highly likely that this error was propagated in derivative works [34].

Data sharing is a key input for COVID-19 models; subsequent sharing of modeling code is equally important. Although it has been said that all models are wrong but some are useful [35], their influence on public policy cannot be ignored [36]. Transparency around model assumptions, parameter estimates, and sources of data and code can allow others to check, build upon, and improve on them as additional information about the virus and its spread becomes available. Although the structure of the models does not appear to have changed since August 2020, Our World in Data provides weekly updates of four major models based on current data [37], which is made available through their code on GitHub [38-41]. Models are produced by scientists, who may or may not have experience in public health policy. Covid Act Now is a distributed team of volunteers including technologists, epidemiologists, public health experts, and public policy leaders working to provide disease intelligence, data analysis, and modeling on COVID-19 for the United States [42].

As the pandemic progressed, larger organizations and citizen scientists created symptom tracking websites such as COVIDNearYou.org and Flatten.ca, the former developed by volunteers from Amazon, Apple, and Google [24], and the latter by Shrey Jain at the University of Toronto [43]. Smartphone and web-based data collection tools have extraordinary potential to accumulate large amounts of data in short periods of time. However, authors in the JMIR COVID-19 Special Issue have expressed a need for a global policy regarding citizen science data collection methods to avoid issues of privacy intrusion and potential harm [44].

### Sharing Data Among Countries in the Americas

Designed to prevent and cope with major international public health threats, 196 countries are signatories to the International Health Regulations. Its stated purpose is “to prevent, protect against, control and provide a response to public health threats through improved surveillance, reporting and international cooperation, and to do so in ways which avoid unnecessary interference with international traffic and trade” [45]. Under Article 44, which deals with collaboration and assistance, the countries of the Americas have shared data for research purposes and to prepare for the potential importation of cases.

The Pan American Health Organization (PAHO), which serves as the Regional Office for the Americas of the WHO and as the health organization of the Inter-American System, facilitates a communication network of national focal points, providing technical cooperation and logistical support for the detection, assessment, and response to events. Between January and November 2020, the countries of the Americas exchanged 643 communications related to the COVID-19 pandemic, particularly to advise other Member States of confirmed cases and contacts during a flight (41%) and confirmed cases and contacts during a cruise or on a ship or vessel (13%), to report travel restrictions or quarantine protocols at points of entry (4%), to request information or verification of an event (9%), to report cases from mass gatherings (2%), for repatriation or evacuations of citizens from one country to another (1%), and for contacts or confirmed cases with or without a travel history (30%).

In addition, since the beginning of the pandemic, the countries of the Americas have reported the daily counts of COVID-19 cases and deaths from official government public sources, which PAHO and the WHO collates, analyzes, and publishes on its regional dashboard [46]. PAHO supports the public health authorities to monitor trends in COVID-19 cases and deaths, identify clusters especially among vulnerable populations, and guide the implementation and adjustment of targeted public health control measures. The dashboard provides geographic distribution of cases and deaths, effective reproductive numbers, and epidemiologic graphs and trends. In-depth analyses on age, gender, testing patterns, and severity is also reported. As of November 6, 2020, details were available for 83% (17,525,352/21,168,405) of total reported cases and 56% (365,540/650,705) of total reported deaths through a shared electronic line list. PAHO also supports countries providing information on the quality of COVID-19 surveillance by monitoring performance indicators such as timelines, completeness, and representativeness of surveillance data. This type of collaboration between Member States and a multilateral organization supports a meaningful interpretation of the surveillance data on a regional basis.

## Crowdsourcing

### The Power of the Crowd

Despite the many parallels between the COVID-19 pandemic and the Spanish flu of 1918, there stands a major distinction: the human population today communicates with unprecedented speed and effectiveness regardless of geographical distance and in the absence of face-to-face contact. This global connectedness has fostered an extraordinary response to the pandemic that vastly outpaces its predecessor just 100 years ago.

Anyone with a connection to the internet could collect information, analyze data, and develop software and hardware. As new needs arose, eager volunteers would band together to identify potential solutions and courses of action. In April 2020, in the course of a week, volunteers from tech giants such as Google, Amazon, and Apple, in collaboration with researchers at Harvard University, Boston Children’s Hospital, and the University of Toronto, had developed “COVID Near You,” an app allowing users to share their symptoms and view the number of potential COVID-19 cases in their community [47]. Early efforts to map the disease by the University of Toronto relied solely on voluntary online self-assessment data [48].

Another call to action was for helping make sense of the accumulating data and drawing meaningful conclusions. Some of the authors of this paper were among the first volunteers to take the raw data being offered by the Government of Canada and translate it into an informational dashboard of preliminary epidemiological analyses interpretable by all [12]. As mentioned in the “Data” section, teams from the University of Toronto in conjunction with the University of Guelph also collaborated in producing a dashboard of critical epidemiological measures [49]. As of January 2021, 28 volunteer-driven data visualization resources have been produced in Canada alone to translate raw data into plots, figures, and tables that can be interpreted by the masses [50]. The collaborative spirit in Canada is demonstrated by the more than 6800 registered volunteers and over 200 volunteer-driven projects (as of January 2021) included in the “COVID-19 Resources” project aimed at matching volunteers nationwide to projects [50].

A larger-scale crowdsourcing effort was undertaken by Google, Apple, and local health authorities in developing contact tracing and exposure notification apps for smartphones [51]. By keeping a record of the contacts a person has had, these notifications can occur retroactively should an individual test or screen positive for the virus. Smartphone contact tracing allows for a secure and constant record of contacts to be kept based on proximity, thus eliminating the need for manual recording. Recognizing the universal need for apps such as these, Google and Apple, rivals in the tech industry, formed a partnership to expedite the software development with the intention that it be adapted and modified by individual public health authorities. Crowdsourcing projects such as these are passive in nature, as they require minimal user input following the initial setup but could play a role in curbing the pandemic with sufficient uptake.

More traditional forms of crowdsourcing, in which a direct invitation to participate is made to the public by an organization, have also been prevalent over the course of the pandemic. Hackathons are competitions in which individuals with experience in software development, design, or other computer-based skills collaborate in a sprint to propose a solution to a challenge. The COVID Global Hackathon, occurring over a 5-day long weekend in March, attracted over 18,000 volunteers from 175 countries, proposing solutions to over 1500 distinct challenges ranging from developing health-monitoring apps for smartwatches to software that connects gamers to fight social isolation [52]. The Montreal General Hospital in collaboration with McGill University challenged volunteers to design a new ventilator, and shortly thereafter, numerous teams produced designs, some at a tenth of the price of traditional machines [53].

The humanitarian efforts were not only limited to designs and theories but also featured the physical production of PPE and machines. Cooper3D, makers of antibacterial 3D printing filaments, recognized the need for alternative forms of respirators, as the global stock of N95s dwindled. Their team designed a 3D model for a new respirator, able to be printed on most at-home and industrial printers, and made the design free to the public [54]. Enthusiasts of 3D printing also jumped on the opportunity to help designing and publishing over 4000 printable files with the tag “COVID-19” to the popular community-driven platform “Thingiverse” in the first 6 months of the pandemic. These files include face masks, door pulls, and air purifiers ready to be printed by anyone with a 3D printer at home [55]. For those with a desire to help but no access to a 3D printer, “Get Us PPE” served as a switchboard, connecting people offering masks (in small or large quantities) or materials to make masks to underresourced communities. The Get Us PPE organization has facilitated the donation of over 5.5 million units of PPE as of January 2021 [56].

As the COVID-19 pandemic persisted, health officials have recognized the health risks of social isolation, and communities have had to adapt to stay connected. Online social communities have boomed as countries such as the United States have seen an increase in social media use by roughly 50% of the population [57]. These online communities not only keep individuals connected but serve as a highway for ideas and information. Reddit users, in just over 3 months, have created the fastest growing community (also known as a “subreddit”) “r/coronavirus,” with over 2 million members voluntarily offering information, support, and high-quality data visualizations [58]. Health officials have warned against misinformation on social media platforms, and through crowdsourcing, this Reddit community has amassed a team of moderators consisting of PhD students, virology experts, and doctors to monitor the tens of thousands of posts a day [59]. Communities of COVID-19 survivors such as “The Survivor Corps” have also emerged, allowing for discussion of experiences to aid health care workers while serving as a support system for the trauma some have endured [60].

### Crowdsourcing and Telemedicine

With border closures, quarantines, and nonpharmaceutical interventions, billions of people are socially isolated. Individuals, governments, and health institutions have turned to information and communication technologies to communicate, work, interact, share information, and generate and exchange knowledge [61]. Telemedicine in particular has proved key to sustaining the continuity of health care services, especially for those with chronic noncommunicable diseases and mental health issues [62].

A crowdsourcing exercise was conducted to obtain goods, services, and ideas for telemedicine from a network of experts across the Americas. The main public good obtained was a “Tool for assessing the maturity level of health institutions to implement telemedicine services” [63], which, although it was designed for the Americas region, was quickly disseminated throughout all continents. It was created by PAHO and the Inter-American Development Bank in collaboration with institutions and experts in telemedicine and the use of information technology in public health from the Region of the Americas and Spain: Italian Hospital of Buenos Aires (PAHO and WHO Collaborating Center for Information Systems and Digital Health), Open University of Catalonia (PAHO and WHO Collaborating Center for eHealth), Center for Health Informatics, University of Illinois (PAHO and WHO Collaborating Center on Information Systems for Health), Salud.uy, Agency for eGovernment and the Information Society of Uruguay, Telemedicine Network from Brazil, the Central American Health Informatics Network, and 10 experts from the PAHO Information Systems for Health network.

The most important result from the crowdsourcing exercise was the transformation of the original idea of a “maturity assessment” into an “accreditation service.” Although this was not originally envisioned, it emanated from debate with experts and national health authorities, and proved to be a powerful aid for governments and institutions that wanted to provide telemedicine services in an effective, legal, and safe manner.

### Crowdsourcing and Artificial Intelligence

Crowdsourcing is a valuable cost-effective tool and, with advancements in the field of AI, citizen scientists have new abilities to help in the global COVID-19 relief efforts. It is no wonder Eric Schmidt (former Google CEO and Executive Chairman of Alphabet Inc) predicted the next US $100 billion dollar company would be one that “uses the crowd to learn,” training an AI platform on crowdsourced data to the point where the AI could outperform the crowd [64].

The purpose of the Fast Healthcare Interoperability Resources (FHIR) data format standard is to make the exchange of electronic health records and health care data easily accessible to both health care providers and individuals on a wide variety of platforms, including computers, tablets, and cell phones [65]. In the face of a global pandemic, the FHIR standard helped facilitate the international exchange of health care data relating to SARS-CoV-2, creating shared open data resources that could be mined by individuals and organizations. For example, the COVID-19 Open Research Dataset Challenge (CORD-19)–on-FHIR data set for COVID-19 research (provided by the Allen Institute for AI to support the ongoing research into COVID-19) was made available for open collaboration through GitHub [66].

The initial data set consisted of over 13,202 journal articles all relating to research on SARS-CoV-2 [67]. Coordinated by The White House Office of Science and Technology Policy, the Allen Institute for AI collaborated with the Chan Zuckerberg Initiative, Georgetown University’s Center for Security and Emerging Technology, Microsoft, and the National Library of Medicine of the National Institutes of Health to expand the data set to over 200,000 scientific articles, all of which were made freely available to the scientific community.

On March 16, 2020, the CORD-19 was launched, challenging experts in the field of AI to answer 17 key questions or “Tasks” that were developed in coordination with the WHO and the National Academies of Sciences, Engineering, and Medicine’s Standing Committee on Emerging Infectious Diseases and 21st Century Health Threats [68]. Using text and data mining, AI experts used the data set to answer key questions such as “What do we know about COVID-19 risk factors?” or “What do we know about diagnostics and surveillance?” A cash prize of US $1000 was awarded for each task to whomever submitted a solution that best met the evaluation criteria set out for each of the 17 questions [67].

## Artificial Intelligence

### Predicting the Spread of a Global Pandemic

With the domestic and international movement of billions of humans and animals annually, the COVID-19 pandemic demonstrated how a local public health event can rapidly grow to become a global emergency. Traditional public health surveillance measures have been historically slow, often relying on reporting from health care facilities being funneled through various channels before information is communicated to the public. Furthermore, the infrastructure required to support these surveillance systems (eg, diagnostic laboratories) has (historically) limited public health surveillance to only the wealthiest of nations [69]. In the current digital era, advanced early warning public health surveillance systems powered by AI offer an alternative. Combining predictive modeling with the capacity to analyze large volumes of data in a variety of formats [70], AI is making public health surveillance faster, cheaper, and smarter.

The Canadian company BlueDot operates at the intersection of AI, public health surveillance, and epidemiology, using machine learning algorithms to collect, synthesize, and assess various data sources, including news reports written in over 60 languages, airline data, and animal disease networks, to detect outbreaks and predict the transmission patterns of disease [71]. Epidemiologists then review and verify outputs from the algorithms, ensuring they are interpretable from an epidemiological perspective. The results are disseminated to a global network of partners consisting of governmental agencies, private industry, and nongovernmental organizations, enabling them to anticipate, prepare, and manage emerging disease threats [71].

At the dawn of the global COVID-19 pandemic, BlueDot was one of the first global entities to detect the emergence of a novel coronavirus (later to be named SARS-CoV-2) in China’s Hubei Province and reported its detection through ProMED-mail on December 30, 2019 [72]. BlueDot proceeded to model the international spread of COVID-19 using various different sources of data from collaborators (eg, flight and passenger travel manifests) to identify the 20 most popular international destinations for flights departing from Wuhan Tianhe International Airport [72]. Using the Infectious Disease Vulnerability Index, BlueDot assessed the capacity for each of those 20 locations to manage an outbreak should the novel coronavirus expand beyond China’s borders and published the first scientific paper on the international spread of COVID-19 [73].

### Predicting Those Most Vulnerable

In addition to powering disease surveillance, AI is enabling new methods to detect those most vulnerable to both emerging and existing health threats. The ClosedLoop.ai platform (powered by Amazon Web Services) uses the C-19 Index, an AI predictive model to detect those most susceptible to COVID-19 [74]. The team behind the C-19 Index developed three different predictive models, each built with different degrees of accuracy and ease of implementation. The objective for each model was the same: predict the likelihood an individual will end up in the hospital within the next 3 months due to COVID-19 [74]. The “Survey” model was the simplest of the three, built using logistic regression and made accessible to the public [75]. The “Open Source” model used gradient boosted trees and had improved accuracy compared to the “Survey” model at the cost of increased user complexity. The source code for this model was uploaded to GitHub, so the health care community could adapt the model to their changing needs [75]. Finally, the “Full” model was the most accurate and most complex of the three models. It too used gradient boosted trees and was built in the ClosedLoop.ai platform and was made available free of charge [75].

## Hardware

### The Private Sector Retools to Meet Demand for PPE

The pandemic has also seen the corporate sector retool and form new partnerships to meet the demands of the crisis. Inksmith was a company that used 3D printing to teach children about topics in science, technology, engineering, and mathematics [76]. Their company responded to a call from the Kitchener-Waterloo Academy of Medicine in Ontario to produce face shields for health care workers. Certification from Health Canada took just 4 days from the time the company pivoted to making face shields [77]. The company produced 1 million face shields by early May 2020 [78], en route to fulfilling an order placed by the Canadian Government for 10 million face shields by the end of August 2020 [79]. Inksmith has partnered with KWArtzLab Makerspace to coordinate a community 3D printing initiative. These additional shields with 3D printed components are donated to teachers, homeless shelters, and other organizations that need PPE [76].

Mustang Survival also retooled itself to address the needs of the pandemic. This marine apparel and goods company manufactured reusable medical gowns in response to the growing need due to COVID-19 [80]. The British Colombian company collaborated with StedFast, a Quebecois textile company, to produce level 3 medical gowns. This partnership was facilitated by Innovation, Science and Economic Development Canada, a branch of the Canadian federal government. Outdoor apparel company Arc’teryx has also joined the effort to produce medical gowns [81]. These two companies expected to produce a total of 90,000 medical gowns.

### Getting a Medical Ventilator Manufactured in Canada

Early in the COVID-19 pandemic, there were concerns that the demand for mechanical ventilators in hospitals would exceed the number available [82]. It would also be difficult to source ventilators, as demand would likely exceed the abilities of existing supply chains. Ventilators for Canadians (V4C) was launched by Canadian businessman Jim Estill of Danby Appliances Inc to address these concerns by organizing a supply chain within Canada [83]. Three other Canadian entrepreneurs joined Estill early in the formation of the V4C consortium: Rick Jamieson of ABS Friction and FTI Professional Grade Inc, Paul L’Heureux of Crystal Fountains, and Scott Shawyer of JMP Solutions [84].

JMP Solutions partnered with Medical Ventilator Milano and Nobel Laureate Arthur McDonald to design a ventilator that could be manufactured in Canada. Medical Ventilator Milano is an international team of scientists and engineers out of Canada, the United States, and Italy [85]. Medical Ventilator Milano itself originated from the Global Argon Dark Matter Collaboration, an international organization looking for an invisible component of the universe known as “dark matter.” A lot of their usual research involves working with gas handling and control systems; thus, Global Argon Dark Matter Collaboration decided to repurpose their expertise to develop additional ventilators to help address the need caused by the pandemic as Medical Ventilator Milano [86]. As part of Medical Ventilator Milano, Dr McDonald led a team of three Canadian labs to develop a ventilator, including Canadian Nuclear Laboratories, TRIUMF, and SNOLAB. Ultimately, JMP Solutions and Medical Ventilator Milano partnered with Vexos Inc to produce the Medical Ventilator Milano Ventilator [87]. The Medical Ventilator Milano Ventilator was approved by the US Food and Drug Administration within just 6 weeks of development.

Meanwhile, FTI Professional Grade Inc partnered with Baylis Medical to develop and manufacture 10,000 ventilators for the Canadian government [88]. These Baylis V4C-560 ventilators are based on the design of the Medtronic PB560 ventilator, which Medtronic made open-source in late March 2020 [89]. Anyone can download, use, and sell ventilators of this design until the WHO’s Public Health Emergency of International Concern ends (or until October 1, 2024) [90]. The first batch of V4C-560 ventilators were approved by Health Canada and were scheduled to arrive in August of 2020 [91].

### Novel Partnerships to Accelerate Test Development and Deployment

The shortage of testing capacity and supplies has been a bottleneck for the global containment of COVID-19. Researchers from industry and academia have been working fervently to develop accurate yet fast, easy, cheap, and scalable tests. On July 28, 2020, the XPRIZE Foundation in conjunction with the nonprofit OpenCovidScreen and a coalition of partners added further incentive by announcing a 6-month competition with US $5 million in prizes to accelerate development of economically viable mass screening tests to enable a safer return to work and school [92,93]. The winning teams’ testing protocols will be documented “in a free, multimedia playbook that will be disseminated globally” [92]. Furthermore, the US $50 million COVID Apollo Project led by life science investors is poised to take these innovations to market [92].

Noninvasive tests (for example, saliva tests that can achieve similar accuracy to testing using nasopharyngeal swabs in people who are asymptomatic) may increase peoples’ willingness to get tested and reduce frontline workers’ risk of viral exposure [94,95]. SalivaDirect is an example that arose from unique circumstances with researchers at Yale University funded by the National Basketball Association and National Basketball Players Association. The test was developed to be agnostic to the equipment on which it is run, having been validated with reagents and instruments from multiple vendors, and its protocol is available as open-source to encourage widespread adoption and production [96,97]. Laboratories around the world can easily obtain the equipment required to carry out the testing without having to rely on proprietary resources. The SalivaDirect protocol is published at protocols.io, a crowdsourced resource where researchers share knowledge and assist each other [98]. The use of a common protocol will also be used to compare data from COVID-19 vaccine candidates within the global laboratory network established by the nonprofit Coalition for Epidemic Preparedness Innovations [99].

## Conclusions

The thread of collaboration, cocreation, and networking that weaved throughout the pandemic response needs to be recognized as more than a series of random events and recorded to inform future efforts, pandemic or nonpandemic related. We have learned to adapt on the fly, incorporating nimbleness and agility into scientific endeavors. New ways of working together, exemplified by the surge in videoconferencing, have been thrust upon us but will have ramifications that last beyond the current pandemic. Our connectedness has allowed us to dynamically share and magnify information about the pandemic, regardless of its reliability. We also recognize that hundreds of millions of people are disconnected and that equity considerations should be a crosscutting strategy for digital transformation in health.

**Figure 1:**
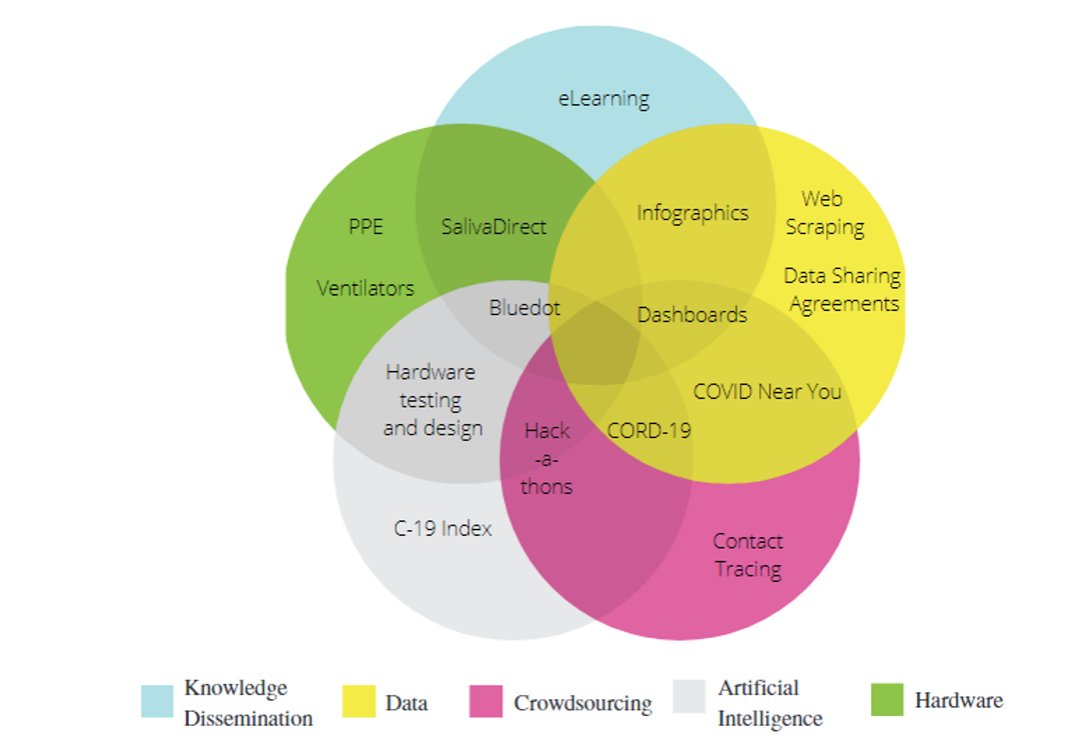
Venn diagram of the five major themes of collaboration with examples. CORD-19: COVID-19 Open Research Dataset Challenge; PPE: personal protective equipment.

The pandemic has spurred the creation of large shared open repositories of health data and research, which have spawned further generations of derivative works. This dependency creates a vulnerability should there be changes or errors in the original source. Innovative processes such as crowdsourcing and competitions have been deployed to populate the repositories and to mine them for answers and solutions, using advanced analytical techniques such as AI. Examples of the intersections among the five major themes can be found in Figure 1.

Novel partnerships, with flexible combinations of citizens, entrepreneurs, small businesses, corporations, academia, and governmental and nongovernmental organizations have crossed national boundaries and disciplinary frontiers to create new processes for working together. They have pivoted and even worked with former competitors to speed development and delivery of equipment and products in short supply. In some cases, they have taken an open-source approach with their solutions, making them available to others to reuse and modify, thus amplifying the end benefits. Born of necessity, it is hoped that this multifaceted progress will be applied not only to the pandemic but also other challenges of global proportion, such as climate change.

## Abbreviations

AI: artificial intelligence
CORD-19: COVID-19 Open Research Dataset Challenge
FHIR: Fast Healthcare Interoperability Resources
PAHO: Pan American Health Organization
PPE: personal protective equipment
V4C: Ventilators for Canadians
WHO: World Health Organization
